# Low-intensity pulsed ultrasound stimulation facilitates *in vitro* osteogenic differentiation of human adipose-derived stem cells via up-regulation of heat shock protein (HSP)70, HSP90, and bone morphogenetic protein (BMP) signaling pathway

**DOI:** 10.1042/BSR20180087

**Published:** 2018-05-22

**Authors:** Zhonglei Zhang, Yalin Ma, Shaowen Guo, Yi He, Gang Bai, Wenjun Zhang

**Affiliations:** 1 Department of Ultrasonography Medicine, Taihe Hospital, Hubei University of Medicine, Shiyan 442000, Hubei Province, China; 2 Department of Obstetrics and Gynecology, Renmin Hospital, Hubei University of Medicine, Shiyan 442000, Hubei Province, China

**Keywords:** adipose-derived stem cells, bone morphogenetic protein, heat shock protein, LIPUS, osteogenic differentiation, ultrasound

## Abstract

Low-intensity pulsed ultrasound (LIPUS) has positive effects on osteogenic differentiation. However, the effect of LIPUS on osteogenic differentiation of human adipose-derived stem cells (hASCs) is unclear. In the present study, we investigated whether LIPUS could promote the proliferation and osteogenic differentiation of hASCs. hASCs were isolated and osteogenically induced with LIPUS stimulation at 20 and 30 mW cm^−2^ for 30 min day^−1^. Cell proliferation and osteogenic differentiation potential of hASCs were respectively analyzed by cell counting kit-8 assay, Alizarin Red S staining, real-time polymerase chain reaction, and Western blotting. The results indicated that LIPUS stimulation did not significantly affect the proliferation of hASCs, but significantly increased their alkaline phosphatase activity on day 6 of culture and markedly promoted the formation of mineralized nodules on day 21 of culture. The mRNA expression levels of runt-related transcription factor, osteopontin, and osteocalcin were significantly up-regulated by LIPUS stimulation. LIPUS stimulation did not affect the expression of heat shock protein (HSP) 27, HSP40, bone morphogenetic protein (BMP)-6 and BMP-9, but significantly up-regulated the protein levels of HSP70, HSP90, BMP-2, and BMP-7 in the hASCs. Further studies found that LIPUS increased the mRNA levels of Smad 1 and Smad 5, elevated the phosphorylation of Smad 1/5, and suppressed the expression of BMP antagonist Noggin. These findings indicated that LIPUS stimulation enhanced osteogenic differentiation of hASCs possibly through the up-regulation of HSP70 and HSP90 expression and activation of BMP signaling pathway. Therefore, LIPUS might have the potential to promote the repair of bone defect.

## Introduction

Many studies have confirmed that mesenchymal stem cells (MSCs) have a strong potential to differentiate into different lineages, such as adipocytes, chondrocytes, osteoblasts, and other nonmesoderm types [1–3]. Traditionally, human MSCs have been isolated from bone marrow, but they can also be isolated from other tissues, such as adipose tissue, fetal liver, umbilical cord blood, and amniotic fluid [4,5]. Recently, as a source of MSCs, human adipose-derived stem cells (hASCs) are a highly attractive source in bone tissue engineering and regenerative medicine because adipose tissue can be easily obtained with less invasive procedures. Furthermore, hASCs display a very high capability for cell proliferation and differentiation [6].

Some studies have indicated that the osteoblast differentiation process of hASCs can be induced by various factors, including soluble molecules, biophysical or chemical signals, and microenvironment [7–9]. Biophysical signals have been actively studied for clinical applications. As a form of mechanical energy, ultrasound stimulation belongs to a biophysical signal that can be conducted into a human body, and results in biochemical events at the cellular level [10]. Low-intensity pulsed ultrasound (LIPUS) has generally been recognized that the micromechanical strains generated by high-frequency acoustic pressure waves evoke biochemical events that can regulate fracture healing [11]. Many studies have demonstrated that LIPUS stimulation produces significant multifunctional effects on bone formation and resorption [12,13]. It has been reported that LIPUS can stimulate fracture healing and has been used to treat bone defects in clinical therapy [14,15], and also accelerates bone maturation in distraction osteogenesis cases in animal models [16].

These previous studies of ultrasound and bone tissue have indicated that LIPUS stimulation has the potential to increase the activity of osteoblasts and to induce the osteogenic differentiation of stem and progenitor cells [17,18]. Although experimental and clinical studies demonstrated the enhancing effects of LIPUS on bone regeneration, to our knowledge, there have been no reports of the effects of LIPUS on the osteogenic differentiation of hASCs and its associated mechanisms. Therefore, the aim of the present study was to investigate the *in vitro* effects of LIPUS on the proliferation and osteogenic differentiation potential of hASCs and preliminarily explored the underlying mechanisms.

## Materials and methods

### Isolation and culture of hASCs

Human subcutaneous adipose tissue samples were taken from three healthy donors during reconstructive surgery. The informed consent was signed by each donor. All procedures were reviewed and approved by the Human Research and Ethical Committee of Hubei University of Medicine. hASCs were isolated as previously described [19]. In brief, subcutaneous adipose tissue was digested with 0.15% collagenase type I (Invitrogen, Grand Island, NY) for 1 h at 37°C. The solution was then filtered through a 70-μm filter to remove undissociated tissues, followed by neutralization by 20% fetal bovine serum (FBS), and centrifuged for 5 min. The stromal cell pellet was resuspended in Dulbecco’s modified Eagle medium (DMEM) containing 10% FBS, 100 U ml^−1^ penicillin, and 0.1 mg ml^−1^ streptomycin, and cultured in a 37°C incubator with 5% CO_2_. After 3 days of culture, the medium was replaced with fresh medium. The adherent cells were maintained in culture and reached confluence, followed by passage to obtain sufficient cells for analysis.

For characterization of the isolated hASCs, the expression of different stem cell markers was analyzed by flow cytometry. The hASCs at passage 3 were incubated with the following primary antibodies: CD13, CD90, CD44, CD105, CD29, CD106, CD31, CD34, CD45, and CD14 (Becton Dickinson, San Jose, CA) for 45 min in the dark, and washed twice with PBS and fixed for 10 min in ice-cold 2% formaldehyde. Flow cytometry was then performed on a FACScan argon laser cytometry (Becton Dickinson) to detect the specific cell surface markers.

### Ultrasound treatment

**Figure 1:**
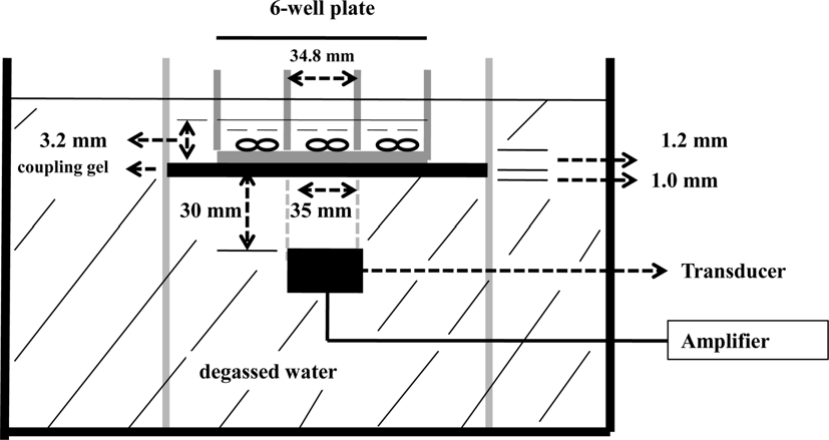
Schematic illustration of ultrasound device The low-intensity pulsed ultrasound device was immersed in a 37°C water bath. The transducer with diameter of 35 mm was positioned using a liner positioning systems, and the level of water was adjusted to ensure an unrestricted acoustic path to the well. A cell culture plate with six wells (diameter = 34.8 mm) was placed on the ultrasound transducer array with a thin layer of ultrasonic coupling gel (1.0 mm). The distance between the transducer and cells was 32.2 mm. The thickness of each well bottom of culture plate was 1.2 mm.

hASCs were seeded into six-well and 96-well plates at an indicated cell density, and cultured in osteogenic medium consisting of DMEM supplemented with 10% FBS, 10 mM β-glycerophosphate disodium, 50 μg ml^−1^ ascorbic acid, and 0.1 μM dexamethasone. After 12-h incubation, the cells were exposed to LIPUS with some modifications, as reported previously [20,21]. Briefly, an ultrasound apparatus (Siemens, Germany) with a resonant frequency of 2.0 MHz was used in the following sonication experiments (Figure 1). The transducer with 35 mm diameter is connected with an ultrasonic generator which works in a continuously adjustable frequency. The transducer surface is circle, and the ultrasound intensity near the transducer surface is roughly constant throughout its cross-sectional area, as reported previously [22]. The transducer was submerged in degassed water, and the culture plates were placed 30 mm above the surface of an array of six transducers with a thin layer of coupling gel. The cells were exposed to ultrasound irradiation following the exposure conditions: a frequency of 2.0 MHz, 200-μs burst width sine wave, a pulse repetition frequency of 1.0 kHz, a pulse duty cycle of 1: 4 (2 ms “on” and 8 ms “off”), a spatial-average temporal-average intensity of 20 and 30 mW cm^−2^ and an exposure time of 30 min daily. The calculated intensity was regarded as spatial averaged and temporal averaged since it measures overall acoustic power without providing spatial and temporal pressure levels [23]. The transfer of proper LIPUS intensity through the water layer to the bottom of each well in the water tank could be confirmed by using the hydrophone and ultrasound power meter before experiment starts. The polystyrene culture plate wells are physically thin (1.2 mm) and are assumed to have a small effect on sound transmission. Ultrasound attenuation within a polystyrene well having thickness (1.22 mm) was reported to be less than 0.3 dB or 4% over the frequency range from 1 to 3 MHz [24]. Additionally, water is a low attenuation medium [24], and ultrasound attenuation through 30 mm degassed water is negligible in the present study. As previously described [25] in a published article, our study can be considered as level 2, based on the nature and quality of ultrasound exposure data. The controls were handled in the same way using separate culture plates without LIPUS treatment. During ultrasound treatment, the temperature in the water bath and culture plates was monitored by a thermocouple as previously described [26], and the temperature in the medium increased from 37 to 40°C during 30 min ultrasound exposure.

### Cell proliferation assay

Cell proliferation was performed as previously described [27], with some modifications. Briefly, hASCs at passage 3 were seeded in 96-well microplates at a density of 1 × 10^3^ cells/well and cultured with or without transient LIPUS stimulation for up to 14 days. The culture medium was replaced with fresh medium every 2 days. On day 0, 2, 4, 6, 8, 10, 12, and 14, a solution of cell counting kit-8 (10 μl) (CCK-8; Genomeditech, Shanghai, China) was added and incubated for 1 h at 37°C. The absorbance values of reaction products were then measured at 570 nm with a microplate reader (ThermoFisher Scientific, Rockford, IL). The relative cell concentration was calculated from the relative absorbance values on the basis of a standard curve.

### Alkaline phosphatase activity assay

Alkaline phosphatase (ALP) activity was determined as previously described [28], with some modifications. In brief, hASCs at passage 4 were seeded in six-well plates at a density of 5 × 10^4^ cells/well, and incubated in osteogenic medium containing DMEM supplemented with 10% FBS, 10 mM β-glycerophosphate disodium, 50 μg ml^−1^ ascorbic acid, and 0.1 μM dexamethasone in the presence or absence of daily LIPUS stimulation for up to 14 days. On day 0, 2, 4, 6, 8, 10, 12 and 14, the cells were lysed with 300 μl solution of a commercial cell lysis kit (ThermoFisher Scientific, Rockford, IL) following the instructions of manufacturer. Twenty microliters of cell lysate was then added into each well of 96-well microplate, followed by addition of 180 μl solution of ALP activity assay kit and 1 mM *p*-nitrophenyl phosphate. The absorbance value of each sample was determined with a microplate reader at 405 nm. The protein content of each sample was measured with a BCA protein assay kit (Beyotime Biotechnology, Haimen, China). The activity of ALP was presented in units per μg protein.

### Real-time polymerase chain reaction (PCR)

hASCs at passage 4 were plated in six-well plates and cultured in osteogenic medium containing DMEM supplemented with 10% FBS, 10 mM β-glycerophosphate disodium, 50 μg ml^−1^ ascorbic acid, and 0.1 μM dexamethasone for 14 days with or without daily LIPUS stimulation. Total RNA was extracted from each sample on day 14 using Trizol reagent (Invitrogen, Carlsbad, USA) following the manufacturer’s instructions, and cDNA synthesis was conducted using a cDNA reverse transcription kit (Applied Biosystems, Foster city, CA). Real-time PCR was performed using SYBR Green I dye (Sigma-Aldrich, U.S.A.) with the following program: 95°C for 5 min; 35 cycles of 95°C for 15 s, and 60°C for 1 min. The sequences of specific primers used in the presence study were as follows: runt-related transcription factor (Runx2), forward: 5′-GCGTCAACACCATCATTCTG-3′, reverse: 5′-CAGACCAGCAGCACTCCATC-3′; osteopontin (OPN), forward: 5′-GTGGTGATCTAGTGGTGCCAAGAGT-3′, reverse: 5′-AGGCACCGGCCATGTGGCTAT-3′; osteocalcin (OCN), forward: 5′-CATGAGAGCCCTCACACTC-3′, reverse: 5′-AGAGCGACACCTAGACCG-3′; Smad 1, forward: 5′-CCAATCTACGAAGGGAGAGTGC-3′, reverse: 5′-AGGGCTTTGGCGGTTGAGTA-3′; Smad 5, forward: 5′-ATAAGAGTTCACCCCGATGC-3′, reverse: 5′-CCAACGTAATCCGTAGTAAGGACA-3′; Noggin, forward: 5′-CCTGAAACAGAAGCGTGGGA-3′, reverse: 5′-ACGCACGAATGGGCTGGT-3′; glyceraldehyde 3-phosphate dehydrogenase (GAPDH), forward: 5′-CAGCGACACCCACTCCTC-3′, reverse: 5′-TGAGGTCCACCACCCTGT-3′. The expression of Runx2, OPN, OCN, and GAPDH was quantified by ΔΔ*C*_T_ methods [29], and their expression levels were normalized to GAPDH mRNA levels.

### Alizarin calcium nodule staining

hASCs at passage 4 were placed into six-well culture plates at a density of 1 × 10^4^ cells/well, and cultured in osteogenic medium containing DMEM supplemented with 10% FBS, 10 mM β-glycerophosphate disodium, 50 μg ml^−^1 ascorbic acid, and 0.1 μM dexamethasone in the presence or absence of daily LIPUS stimulation for up to 21 days, followed by fixation in 70% ethanol for 30 min, and stained with 2% Alizarin Red S (Shanghai Haoran company, Shanghai, China) at pH 4.0 for 5 min. After washing with PBS twice, the cells and calcium nodule formation were checked and captured under phase contrast microscopy with a digital camera (Nikon, Tokyo, Japan).

### Western blot analysis

hASCs at passage 4 were seeded into six-well microplates at a density of 5 × 10^4^ cells/well, and cultured in osteogenic medium containing DMEM supplemented with 10% FBS, 10 mM β-glycerophosphate disodium, 50 μg ml^−1^ ascorbic acid, and 0.1 μM dexamethasone for indicated time points in the presence or absence of LIPUS stimulation, and the cells were lysed in the lysis buffer containing a protease inhibitors mixture. After determination of protein concentrations with a Bradford assay (Bio-Rad, Hercules, CA), equal amounts of total protein (10 μg each sample) were loaded onto 10% Tris-glycine gels, and electrophoresis was performed for 40 min. Then, the separated proteins were transferred to polyvinylidene difluoride membranes, followed by block with TBST (10 mmol l^−1^ Tris-HCl pH 7.5, 150 mmol ml^−1^ NaCl, and 0.1% Tween-20) buffer containing 5% nonfat milk for 1 h before incubation with primary antibodies against bone morphogenetic protein (BMP)-2, BMP-6, BMP-7, BMP-9, heat shock protein (HSP)27, HSP40, HSP70, HSP90, Runx2, OPN, OCN, p-Smad 1/5, Smad 5, Noggin, and β-actin (Cell Signal Technology, Beverly, MA, USA) for overnight at 4°C, followed by the addition of biotin-conjugated secondary antibodies. The membranes were washed with TBST buffer, followed by incubation with horseradish peroxidase-conjugated streptavidin, and then the immunoreactive proteins were detected with an enhanced chemiluminescence analysis system (Amersham Life Science, Arlington Heights, IL).

### Statistical analysis

All data were presented as the mean ± standard deviation (SD). Significant differences were performed by one-way ANOVA followed by Tukey’s multiple comparisons post-test using SPSS statistical software package (version 12.0). A value of *P*<0.05 was considered statistically significant.

## Results

### Characterization of hASCs

**Figure 2:**
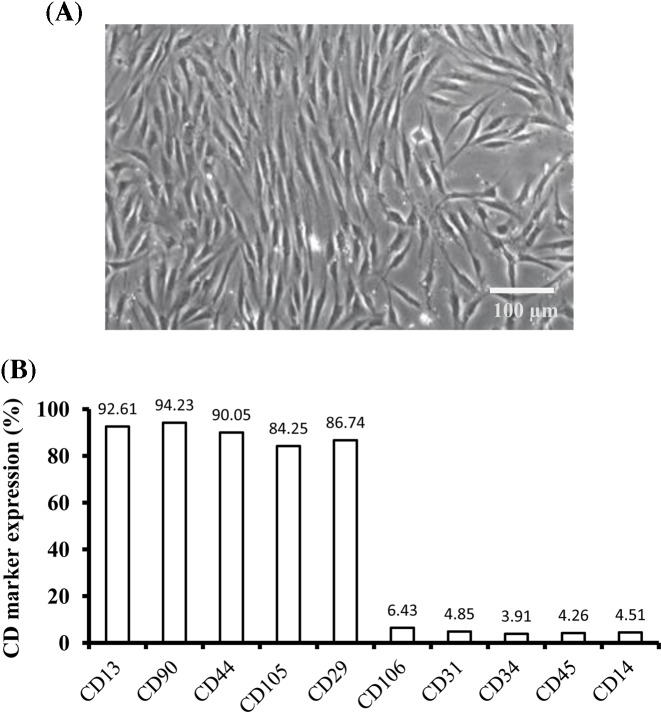
Morphology and immunophenotype of hASCs (**A**) The isolated hASCs at passage 2 displayed fibroblast-like shape attached on culture flasks. (**B**) The mean percentage of positively stained cells as detected by flow cytometry.

Human primary ASCs at passage 2 from subcutaneous adipose tissues exhibited typical fibroblast-like morphology and attached on the flasks (Figure 2A). The results of flow cytometry showed that these hASCs at passage 3 expressed high levels of cell surface antigens of CD29 (92.61%), CD90 (94.23%), CD44 (90.05%), CD105 (84.25%), and CD29 (86.74%), but with low levels of CD106 (6.43%), CD31 (4.85%), CD34 (3.91%), CD45 (4.26%), and CD14 (4.51%) (Figure 2B), consistent with the mesenchymal stem cells obtained from bone marrow.

### Effect of LIPUS stimulation on ASCs proliferation

**Figure 3:**
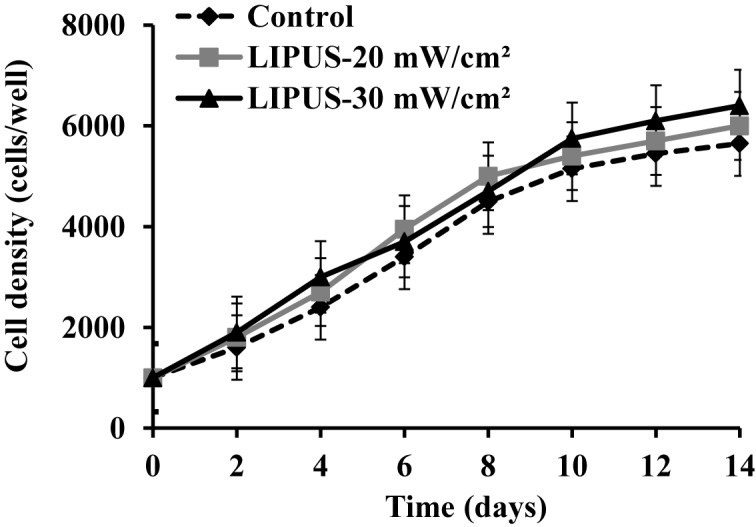
Effect of LIPUS stimulation on cell proliferation hASCs were cultured with or without transient LIPUS stimulation and the cell numbers were determined on day 2, 4, 6, 8, 10, 12, and 14 of culture. The data are shown as the mean ± SD of three separate experiments.

The proliferation of hASCs was measured in the presence or absence of transient LIPUS stimulation for up to 14 days of culture. As shown in Figure 3, exposure to ultrasound stimulation at 20 and 30 mW cm^−2^ did not produce a significant impact on cell proliferation compared with the control cells (*P*>0.05).

### Effect of LIPUS stimulation on alkaline phosphatase activity

**Figure 4:**
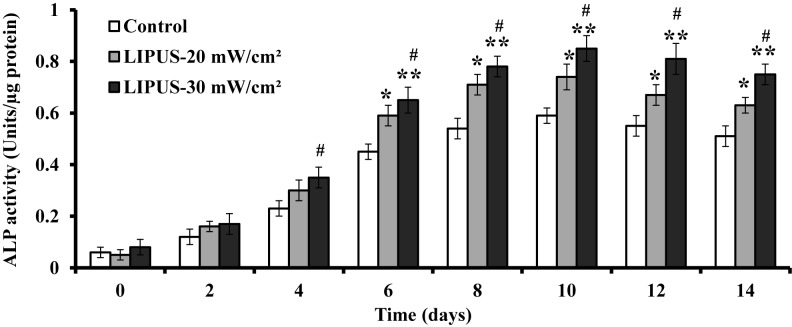
Effect of LIPUS stimulation on alkaline phosphatase activity hASCs were daily exposed to LIPUS stimulation at 20 and 30 mW cm^−2^ for 30 min, and the activity of ALP was determined on day 2, 4, 6, 8, 10, 12, and 14 of culture. The data represented as mean ± SD (*n*=3); *P<0.05 and **P<0.01 versus control; ^#^*P* < 0.05 versus LIPUS, 20 mW cm^−2^.

The activity of ALP was detected in the ASCs stimulated with LIPUS at 20 and 30 mW cm^−2^ for 30 min daily. The results indicated that ALP activity gradually increased through 10 days of culture regardless of LIPUS stimulation, and it slightly decreased on day 12 and 14. Further analyses demonstrated that ALP activity increased by 15% and 20% on day 6, 17% and 24% on day 8, 14% and 26% on day 10, 13% and 25% on day 12, 12% and 24% on day 14 when the cells were respectively treated with LIPUS at 20 and 30 mW cm^−2^, as compared with the corresponding control. Therefore, ALP activity was significantly higher in the LIPUS-stimulated cells than that in the control cells on day 6, 8, 10, 12, and 14 of culture (Figure 4).

### Osteogenesis-related gene expression

**Figure 5:**
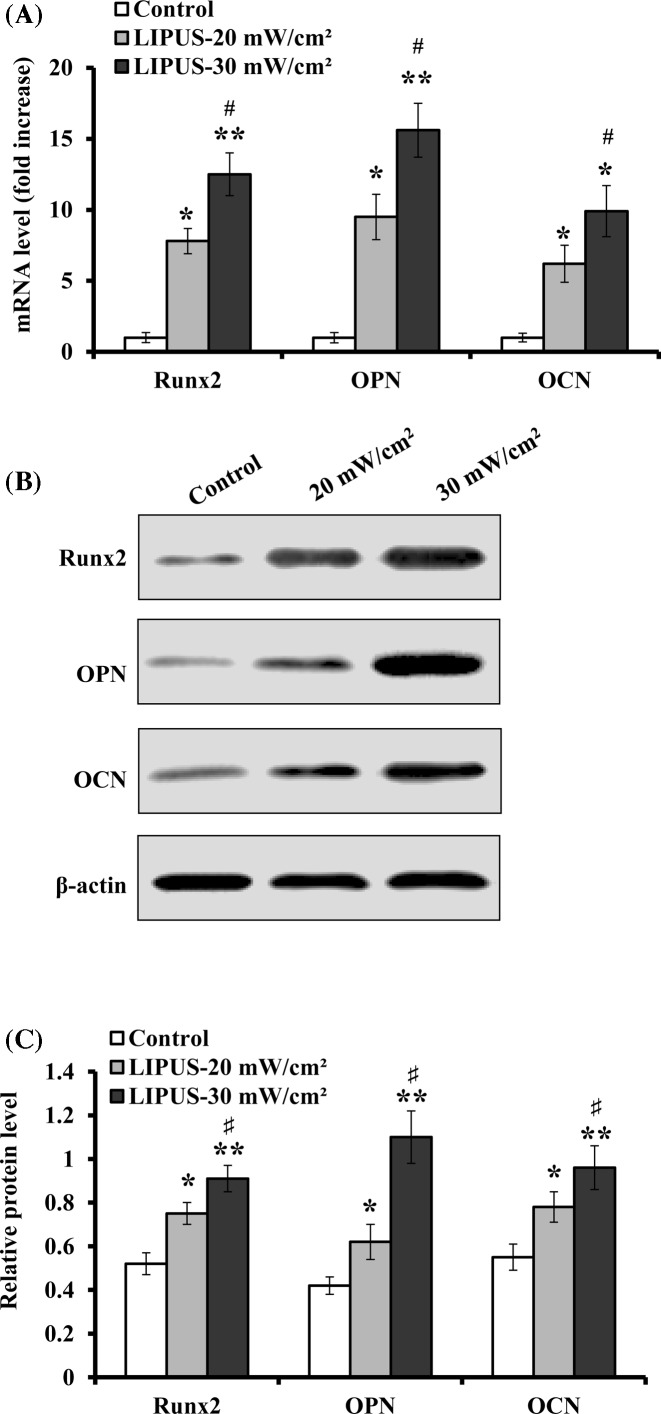
Expression levels of Runx2, OPN, and OCN during ASCs osteogenic differentiation hASCs were treated with LIPUS at 20 and 30 mW cm^−2^ for 30 min daily, and the expression of Runx2, OPN, and OCN was analyzed on day 14. (**A**) The mRNA levels of Runx2, OPN and OCN were determined by real-time PCR, and normalized to GAPDH, and presented as fold increase relative to the controls (value set at 1). (**B**) Representative images of Western blotting. (**C**) The protein levels of Runx2, OPN, and OCN were quantified by normalization to β-actin. Data are presented as mean ± SD (*n*=3); *P<0.05 and **P<0.01 versus control; ^#^*P*<0.05 versus LIPUS, 20 mW cm^−2^.

The effect of LIPUS stimulation on osteogenesis-related gene expression such as Runx2, OPN, and OCN was evaluated by real-time PCR and Western blot analysis. The results demonstrated that the mRNA expression levels of Runx2, OPN, and OCN increased by 6.8 ± 0.9 and 11.5 ± 1.5 fold, 8.5 ± 1.6 and 14.6 ± 1.9 fold, 5.2 ± 1.3 and 8.9 ± 1.8 fold, respectively, in comparison with the control after 14 days of culture with LIPUS stimulation at 20 and 30 mW cm^−2^ for 30 min daily (Figure 5A). Additionally, the protein levels of Runx2, OPN, and OCN were all significantly up-regulated, and showed a consistent increase trend with their mRNA levels (Figure 5B,C). Furthermore, LIPUS intensity at 30 mW cm^−2^ resulted in stronger effect on the expression of Runx2, OPN, and OCN.

### Mineralized nodule formation

**Figure 6:**
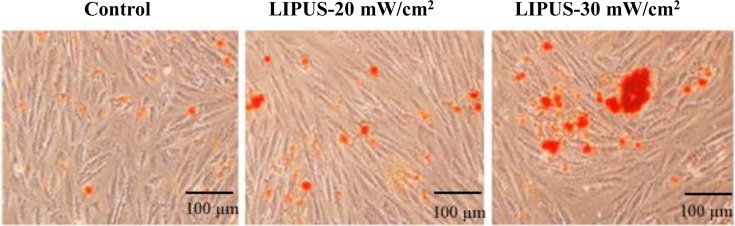
Effect of LIPUS stimulation on mineralized nodule formation hASCs were stimulated with LIPUS at 20 and 30 mW cm^−2^ for 30 min day^−1^ for 21 days, and the formation of calcium nodule was evaluated by staining with Alizarin Red S.

Mineralized nodule formation was evaluated on day 21 of culture with or without LIPUS exposure at 20 and 30 mW cm^−2^ for 30 min daily. As shown in Figure 6, the result exhibited that ultrasound treatment increased the formation of mineralized nodules, as displayed more intense Alizarin Red S staining in LIPUS-treated cells than in control cells. These data suggest that ultrasound stimulation enhanced the osteogenic differentiation of hASCs.

### Effects of LIPUS on the expression of HSPs and BMPs

**Figure 7:**
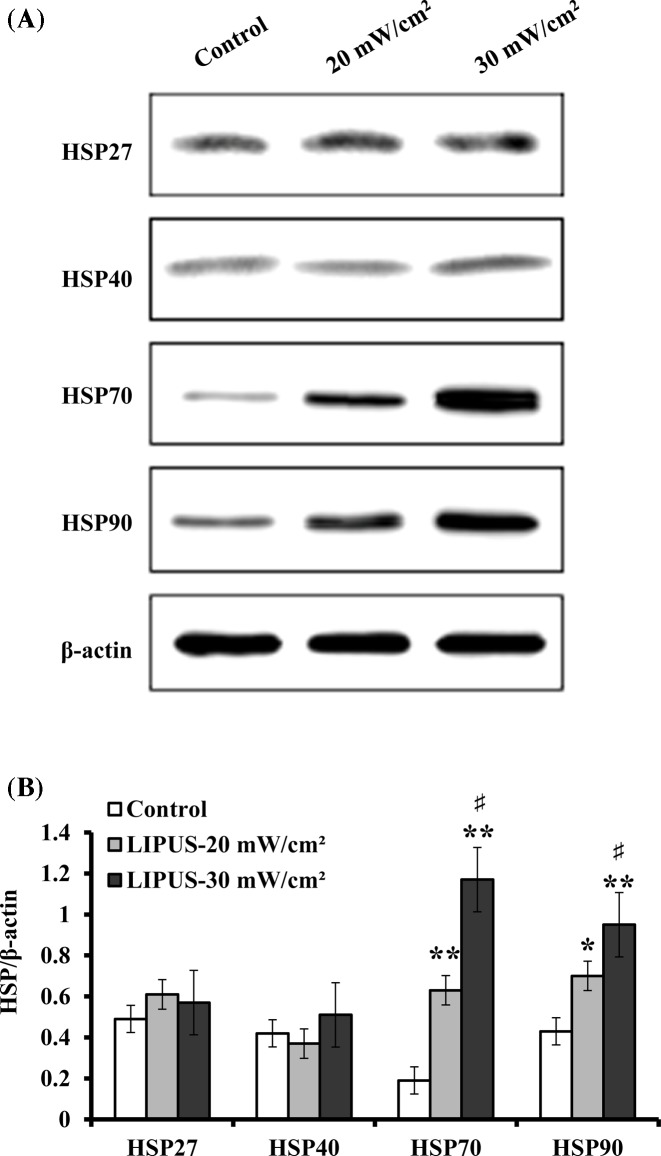
Analyses of HSP27, HSP40, HSP70, and HSP90 hASCs were treated with LIPUS at and 30 mW cm^−2^ for 30 min, and the temperature in the culture medium increased from 37 to 40°C during 30 min ultrasound exposure. Cell lysates were then extracted and subject to SDS/PAGE analysis. (**A**) Representative Western blot bands that exhibited the protein expression of HSP27, HSP40, HSP70, and HSP90. (**B**) The protein levels of HSP27, HSP40, HSP70, and HSP90 relative to β-actin that was used as a loading control. Data are presented as mean ± SD (*n*=3); *P<0.05 and **P<0.01 versus control; ^#^*P*<0.05 versus LIPUS, 20 mW cm^−2^.

Previous studies have suggested that there is a synergistic interaction between heat and ultrasound [26], and LIPUS stimulation can up-regulate HSP90 expression in mouse calvaria-derived osteoblasts [20]. Therefore, we decided to detect the protein levels of HSP27, HSP40, HSP70, and HSP90 using Western blot analysis. The results demonstrated that LIPUS stimulation did not markedly affect the levels of HSP27 and HSP40, but HSP70 and HSP90 were found to significantly increase by 211% and 506%, 52% and 113% for the cells treated with 20 and 30 mW cm^−2^ for 30 min day^−1^ for 14 days, respectively, as compared with the untreated cells (Figure 7).

**Figure 8:**
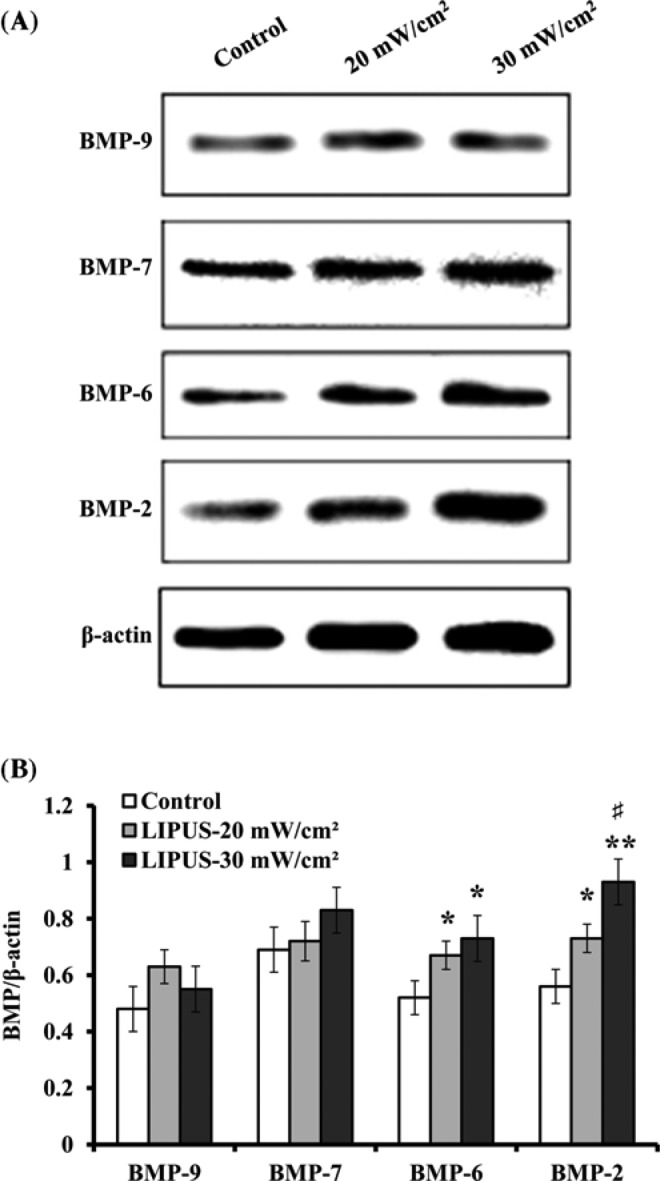
Effect of LIPUS stimulation on BMPs hASCs were treated by LIPUS at 20 and 30 mW cm^−2^ for 30 min daily, and the protein levels of BMP-2, BMP-6, BMP-7, and BMP-9 were detected using Western blot analysis. (**A**) Representative Western blot bands that displayed the protein expression of BMP-2, BMP-6, BMP-7, and BMP-9. (**B**) The protein levels of BMP-2, BMP-6, BMP-7, and BMP-9 relative to β-actin used as a loading control. Data are presented as mean ± SD (*n*=3); *P<0.05 and **P<0.01 versus control; ^#^*P*<0.05 versus LIPUS, 20 mW cm^−2^.

On the other hand, BMPs have been shown to be an important mediator in the ultrasound-mediated osteoblast differentiation [30]. Accordingly, the protein levels of BMP-2, BMP-6, BMP-7, and BMP-9 were analyzed after exposure to LIPUS at 20 and 30 mW cm^−2^ for 30 min day^−1^ for 14 days. As shown in Figure 8, no significant differences in the expression levels of BMP-6 and BMP-9 proteins were found between ultrasound-treated and untreated cells. However, the protein levels of BMP-2 and BMP-7 were observed to be up-regulated by 18% and 30%, 20% and 56% for the cells treated with LIPUS stimulation at 20 and 30 mW cm^−2^ for 30 min day^−1^ for 14 days, respectively, as compared with the untreated cells.

### Evaluation of BMP signaling

**Figure 9:**
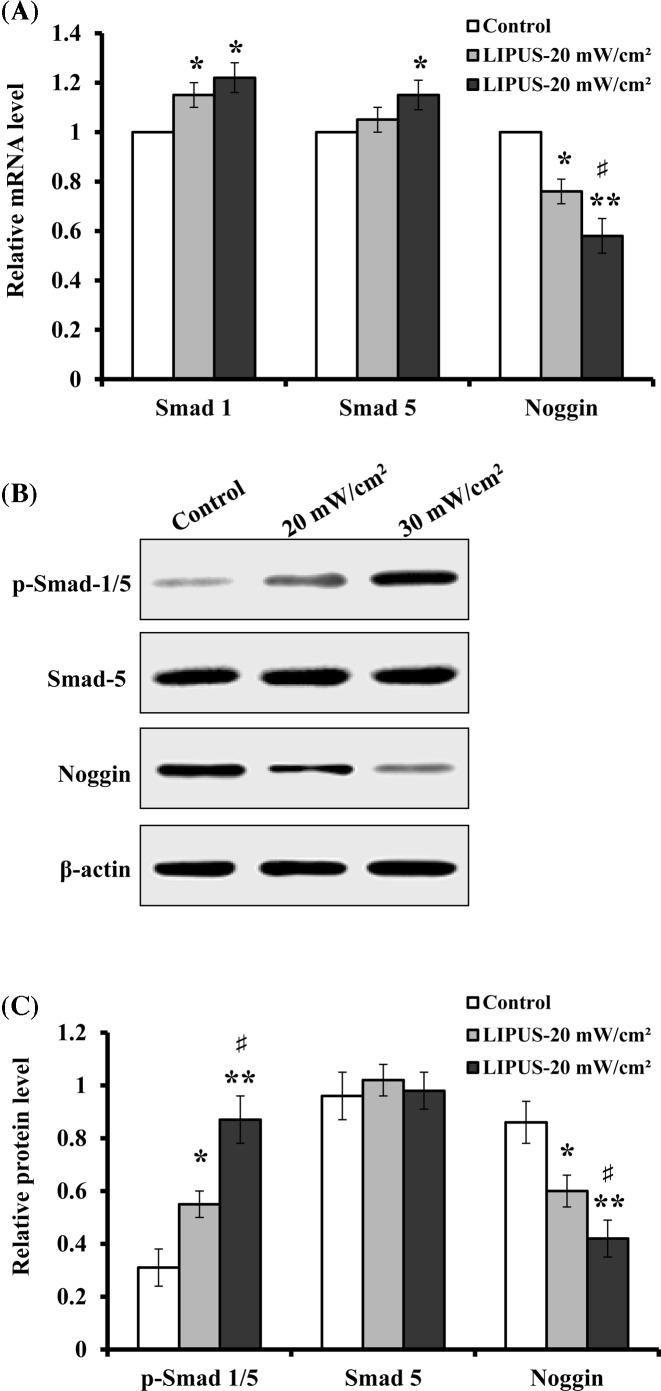
Evaluation of BMP signaling and Noggin expression hASCs were treated with LIPUS at 20 and 30 mW cm^−2^ for 30 min daily, and on day 14, the expression of BMP downstream genes Smad and antagonist Noggin was evaluated. (**A**) Real-time PCR was used to determine the mRNA levels of Smad 1, Smad 5, and Noggin. (**B**) Representative images of Western blotting. (**C**) The protein levels of p-Smad 1/5, Smad5, and Noggin were determined by normalization to β-actin. Data are presented as mean ± SD (*n*=3); *P<0.05 and **P<0.01 versus control; ^#^*P*<0.05 versus LIPUS, 20 mW cm^−2^.

The regulation of BMP signaling has been found to be important in osteoblast differentiation and adult skeletogenesis [31]. Therefore, we determined the expression levels of BMP antagonist Noggin and BMP downstream genes Smad 1 and Smad 5. The results of qRT-PCR analysis demonstrated that the mRNA levels of Smad 1 and Smad 5 increased by 16% and 22%, 5% and 14% for the cells treated with LIPUS at 20 and 30 mW cm^−2^, respectively, when compared with the untreated cells, but Noggin expression was down-regulated by 25% and 42% (Figure 9A). The results of Western blot analysis showed that LIPUS stimulation induced a detectable increase in phosphor-Smad 1/5 (p-Smad 1/5) relative to the control cells when the cells treated with 20 and 30 mW cm^−2^. Total Smad 5 protein level was not found to exhibit any considerable change (Figure 9B). The protein level of Noggin showed a similar trend with its mRNA level. Further analysis indicated that its protein level decreased by 30% and 51%, respectively, as compared with the controls when the cells were treated for 30 min day^−1^ for 14 days with 20 and 30 mW cm^−2^ (Figure 9C).

## Discussion

LIPUS has been widely used as an efficient and safe therapeutic approach in bone regeneration [32], and it is a form of mechanical energy which is transmitted into body tissues as an acoustic pressure wave, producing biochemical events at the cellular level [33]. Some previous studies demonstrated that the osteoinductive response *in vitro* and the acceleration of bone regeneration *in vivo* induced by LIPUS were through its nonthermal effects [34,35]. However, the exact use of ultrasound stimulators is still controversial because of side effects, and its biophysical mechanisms involved in the fracture healing process need to be further studied. In the present study, our data indicated LIPUS stimulation at the intensity of 20 or 30 mW cm^−2^ for 30 min day^−1^ resulted in a significant increase in ALP activity and the formation of mineralized nodules in the hASCs. The up-regulated mRNA and protein expression levels of Runx2, OPN, and OCN were observed in the hASCs after LIPUS treatment. These data indicate that LIPUS stimulation enhanced the osteogenic differentiation of hASCs *in vitro*, and also suggest that LIPUS might have a positive application in the clinical treatment of bone fracture through bone regeneration. It has demonstrated that the stimulation of bone healing was a result of an increase in LIPUS-induced intracellular Ca^2+^ that occurs within seconds after LIPUS stimulation [36].

hASCs is a promising autologous cell source for bone tissue engineering and regenerative medicine, mainly due to its wide availability and easy access [37]. The acceleration of cell differentiation can be marked by increased ALP activity, which is an early marker of osteoblast maturation and differentiation [22]. ALP is a hydrolase enzyme which not only hydrolyzes substances suppressing calcification, but is also necessary for producing the increased phosphate concentration required for hydroxyapatite crystallization [38]. Therefore, ALP plays an important role in the calcification of bone. In the present study, a significant increase in ALP activity in the hASCs was observed after 6 days of stimulation by LIPUS at 20 and 30 mW cm^−2^ for 30 min daily. These findings are similar with previous reports that the ALP activity in osteoblasts or osteoblast-like cells began to increase from day 5 after daily transient LIPUS treatment, indicative of enhanced osteogenesis [39]. In addition, ALP activity reached a peak on day 10 with LIPUS stimulation, followed by a slight drop until day 14, when late-stage osteogenic differentiation of hASCs perhaps occurred, suggesting that the cells had gone past the stage where ALP was expressed maximally. These findings indicate that transient LIPUS treatment may enhance mineralization and stimulate differentiation of hASCs into the osteogenic lineage by increasing ALP activity.

To further confirm LIPUS-induced osteogenic differentiation of hASCs, the expression of osteogenesis-related markers such as Runx2, OPN, and OCN was evaluated by real-time PCR and Western blot analysis. We found that LIPUS treatment resulted in a significant up-regulation in the mRNA and protein expression levels of Runx2, OPN, and OCN in hASCs, as compared with nontreated cells. Other studies have confirmed the influence of LIPUS on the expression of Runx2, OPN, and OCN [40,41]. Runx2 is a master regulatory transcription factor for osteogenesis, and it is essential for osteoblastic differentiation and early up-regulation in the osteoblast differentiation process [42]. Activation of Runx2 activation increases osteocalcin and collagen Ι gene expression [43], and Runx2 is considered to be the target of a mechanical signal by which physical stimulation dictates the cellular and metabolic activities of osteoblasts [44]. Additionally, OPN is a highly acidic secreted phosphoprotein, and can induce differentiation of osteoblasts and promote bone tissue remodeling [45]. Osteocalcin is also thought to play a role in bone-building and bone mineralization, and it is often used as a late-stage marker of osteogenic differentiation and bone formation process [46], which is in agreement with our findings that the mineralized nodule formation upon LIPUS stimulation increased in hASCs. Taken together, our findings provide molecular evidence to explain LIPUS-promoted osteogenic differentiation of hASCs.

HSPs are ubiquitous and highly conserved proteins in all organisms under various stress conditions, and their synthesis can be initiated not only by heat shock, but also by many physical and chemical stimuli, such as heavy metals, oxidative stress, amino acid analogues, UV, and ionizing irradiation [47]. HSPs play fundamental roles in many pathophysiologic and physiologic processes, such as degradation of unstable proteins, control of regulatory proteins, and import and folding of proteins [48]. HSPs are also associated with bone metabolism. It was reported that HSP27 regulated the balance between the differentiation and apoptosis of osteoblasts [49]. HSP90 expression was up-regulated by LIPUS stimulation in mouse calvaria-derived osteoblasts [20]. In the present study, we found that the protein levels of HSP70 and HSP90 were significantly up-regulated by LIPUS treatment, although HSP27 and HSP40 expression was not changed in the hASCs. Previous studies showed that although acute ultrasound exposure did not induce HSP70 [50], a series of four exposures did result in increased production of HSP70 [51]. Additionally, mechanical stress, such as heat or LIPUS, probably does not induce the low-molecular weight HSP27 and HSP40, but it instead activates the high-molecular weight HSP90 that acts as a molecular chaperone or protects cells subjected to hazardous conditions [20]. However, it remains to be confirmed in future investigation whether HSPs induction is a beneficial or detrimental response during exposure to LIPUS stimulation. Evidence of embryonic stress induced by pulsed ultrasound combined with a modest temperature increase is shown by retarded development and heat shock protein synthesis in rat embryos [52]. Therefore, insight into the exact role of HSPs may have important clinical implications in administration of LIPUS because HSPs induction might be adjusted in LIPUS duration or intensity to minimize or maximize HSPs expression. Additionally, a temperature rise from 37 to 40°C was observed during 30 min ultrasound exposure in the present study. Temperature elevation is able to induce HSPs expression, which suggests temperature increase by 3°C can be detected by the cells and results in reactions at the subcellular level. Temperature rise probably resulted from ultrasound absorption and conduction from the transducer. As previous reported, the amount of ultrasound-induced temperature increase occurring during an ultrasonic exposure depends on the properties of both the ultrasound field parameters and the biological tissue involving ultrasound absorption, thermal conduction, and blood perfusion [52]. Previous studies have indicated that BMPs have been shown to be important mediators for regulating osteoblast differentiation [53], and LIPUS treatment enhances BMP-7-induced osteogenic activity of nonunion tissue-derived cells [54]. In the present studies, we found that the expression levels of BMP-2 and BMP-7 were significantly increased by LIPUS treatment on hASCs, although BMP-6 and BMP-9 expression was not significantly changed. BMP-2 was found to induce an increase in ALP activity in MC3T3-E1 osteoblastic cells, and significantly stimulated collagen synthesis [55]. LIPUS enhanced recombinant human BMP-2-induced ectopic bone formation in a rat model [56], and increased BMP-2 mRNA expression in rat osteoblasts [21]. Additionally, BMP-7 is a strong stimulator of osteogenic differentiation, and its induction on osteogenic activity of nonunion tissue-derived cells is increased by LIPUS [54]. On the basis of our findings, it seems possible that LIPUS stimulation combined with application of recombinant human BMP-2 and BMP-7 may accelerate fracture healing.

BMPs exert their activity by binding to their receptors on the cell surface, and transduce signals by activating their downstream Smad proteins [57]. Noggin, a BMP antagonist acts by directly binding to the BMP and preventing BMPs from binding to their receptors [58]. In the present study, we found that LIPUS significantly down-regulated Noggin expression at the end of 14 daily treatment. The intensity of 30 mW cm^−2^ showed a stronger inhibitory role on Noggin expression than another treatment group (20 mW cm^−2^). Therefore, these findings suggest that Noggin inhibition contributes to LIPUS-mediated osteogenic differentiation of hASCs. Furthermore, our results showed that 30 mW cm^−2^ intensity produced better effect on osteogenic differentiation of hASCs than 20 mW cm^−2^. Previous studies have indicated that Noggin suppression can enhance *in vitro* osteogenesis of ASCs and accelerate *in vivo* bone formation [59,60]. Additionally, we analyzed the transcription of Smad 1 and Smad 5 by qRT-PCR. Relative to untreated ASCs, the transcription levels of Smad 1 and Smad 5 showed an increase at the end of 14 daily LIPUS treatment, but no change was observed in total Smad 5 protein level, which demonstrated that Noggin reduction up-regulated mRNA levels of these BMP signaling Smad intermediates [59]. A significant increase in phosphor-Smad 1/5 was observed relative to control cells. These findings indicate that LIPUS stimulation enhances the signaling activity of BMPs, as reflected by the decrease in Noggin expression and the increase in phosphorylated Smad intermediates.

In conclusions, our findings demonstrated that LIPUS stimulation increased ALP activity in hASCs, and promoted mineralized nodule formation, and up-regulated the expression of Runx2, OPN, and OCN. These findings suggest that LIPUS treatment enhances the osteogenic differentiation of hASCs, and the LIPUS-mediated mechanism of osteogenic differentiation may be achieved via the up-regulation of HSP70 and HSP90 expression and activation of BMP signaling pathway in the hASCs. Our results provide significant evidence for the potential usefulness of the clinical application of LIPUS to accelerate fracture healing. It is likely that LIPUS has an important influence on key functions activities of ASCs in bone regeneration. It should be noted that these *in vitro* findings cannot be extrapolated to *in vivo* conditions directly, and further studies in an animal model are needed to elucidate the roles of LIPUS stimulation on osteogenic differentiation of hASCs.

## Abbreviations

ALP: alkaline phosphatase
BMP: bone morphogenetic protein
DMEM: Dulbecco’s modified Eagle medium
FBS: fetal bovine serum
GAPDH: glyceraldehyde 3-phosphate dehydrogenase
hASC: human adipose-derived stem cell
HSP: heat shock protein
LIPUS: low-intensity pulsed ultrasound
MSC: mesenchymal stem cell
OCN: osteocalcin
OPN: osteopontin
PBS: phosphate buffer saline
Runx2: runt-related transcription factor
